# Mindfulness-Based Stress Reduction (MBSR) as a Standalone Intervention for Posttraumatic Stress Disorder after Mixed Traumatic Events: A Mixed-Methods Feasibility Study

**DOI:** 10.3389/fpsyg.2017.01407

**Published:** 2017-09-05

**Authors:** Meike Müller-Engelmann, Susanne Wünsch, Marina Volk, Regina Steil

**Affiliations:** ^1^Department of Clinical Psychology and Psychotherapy, Institute of Psychology, Goethe University Frankfurt Frankfurt, Germany; ^2^Trauma-und Opferzentrum Frankfurt e.V. Frankfurt, Germany

**Keywords:** posttraumatic stress disorder, depression, MBSR, mindfulness, treatment

## Abstract

**Objectives:** There is promising evidence that mindfulness-based interventions are effective in reducing the symptoms of posttraumatic stress disorder (PTSD). However, until now, studies have often lacked a full clinical PTSD assessment, and interventions are often administered in addition to other interventions. This study examined the feasibility of mindfulness-based stress reduction (MBSR) as a standalone intervention in patients with PTSD who have experienced mixed traumatic events.

**Method:** Fourteen patients participated in 8 weeks of MBSR. The patients were assessed prior to treatment, post-treatment and at a 1-month follow-up through self-ratings (e.g., the Davidson Trauma Scale) and the Clinician-Administered PTSD Scale to determine the effects of the intervention. Furthermore, after the intervention, the patients participated in qualitative interviews regarding their experiences with MBSR and their ideas for future improvements.

**Results:** Nine patients finished the program, and these patients considered the exercises to be applicable and helpful. In the Clinician-Administered PTSD Scale, we found large effects regarding the reduction of PTSD symptoms among completers (Cohen's *d* = 1.2). In the Davidson Trauma Scale, the effect sizes were somewhat lower (Cohen's *d* = 0.6) but nevertheless confirmed the efficacy of MBSR in reducing PTSD symptoms. In the qualitative interviews, the patients reported an augmentation of wellbeing and improvement regarding the handling of difficult situations and more distance from the traumatic event.

**Conclusion:** Despite the large effects, the high dropout rates and the results of the post-treatment interviews suggest that the intervention should be better adapted to the needs of PTSD patients, e.g., by giving more information regarding the exercises and by including shorter exercises to manage acute distress.

## Introduction

A traumatic event is defined as exposure to actual or threatened death, serious injury or sexual violence (American Psychiatric Association., 2013). Approximately 25% of people who have experienced a traumatic event later develop clinically relevant symptoms of posttraumatic stress disorder (PTSD) that require treatment (Hidalgo and Davidson, 2000). With a lifetime prevalence of 6.8%, PTSD is an important health care problem (Kessler et al., 2005). PTSD comprises symptoms of intrusion, avoidance, negative alterations in cognitions and mood, and alterations in arousal and reactivity (American Psychiatric Association., 2013).

Meta-analyses regarding PTSD treatment demonstrate the efficacy of trauma-focused interventions (Watts et al., 2013; Cusack et al., 2016), such as prolonged exposure (Foa et al., 2007), cognitive processing therapy (Resick and Schnicke, 1993), and eye movement desensitization and reprocessing (Shapiro and Forrest, 2001). However, clinical experience indicates that many severely affected patients are unwilling to directly address the traumatic event; therefore, compliance with trauma-focused treatment is often low (Scott and Stradling, 1997), non-response rates are high (Schottenbauer et al., 2008), and a substantial number of patients still have residual symptoms after treatment (Bradley et al., 2005). Furthermore, there are often high dropout rates in PTSD treatments (Imel et al., 2013), sometimes up to 54% (Schottenbauer et al., 2008). An additional difficulty is that exposure-based techniques are underutilized in clinical practice and lack acceptance because of therapists' concerns regarding problematic behaviors, such as suicide attempts or substance abuse (Becker et al., 2004).

Accordingly, there is a need to develop further interventions for treating PTSD to ensure that more people can be reached and treated effectively. Mindfulness-based interventions are a group of interventions that have recently received attention in the context of PTSD treatment (Banks et al., 2015). Mindfulness is characterized by bringing one's attention to the experience of the present moment with a non-judgmental attitude (Kabat-Zinn, 2013). One reason for the assumption that mindfulness-based interventions may help to reduce PTSD is that re-experiencing symptoms are thought to be maintained by perceiving the trauma as a current threat (Ehlers and Clark, 2000) and that mindfulness practice through an awareness of the present moment may improve the ability to distinguish between past and present and by this reduce intrusions. Furthermore, the avoidance of distressing memories is considered to be crucial to the maintenance of PTSD (O'Donnell et al., 2007). Studies show that avoidance leads to an increase of intrusions (Wegner and Zanakos, 1994; Steil and Ehlers, 2000), trauma-related thoughts (Shipherd and Beck, 2005) and associated emotions (Beck et al., 2006). Training in mindfulness may thus reduce PTSD symptoms by increasing the ability of patients to tolerate contact with distressing memories, thoughts and feelings (Follette et al., 2006). Furthermore, PTSD patients often suffer from negative thoughts, feelings and related physical reactions (American Psychiatric Association., 2013), and mindfulness may help to identify and stop at an early stage dysfunctional, mutually reinforcing processes of, e.g., emotional and physical reactions (Baer, 2003). Correspondingly, one study found non-reactivity to inner experiences to be associated with lower PTSD symptoms (Kalill et al., 2014). Additionally, practicing mindfulness may decrease PTSD-related hyperarousal, because the exercises produce stress reduction and relaxation effects (Chiesa and Serretti, 2009), which has been demonstrated by a lower cortisol level at awakening in PTSD patients after mindfulness meditation (Wahbeh et al., 2016).

The idea that mindfulness-based interventions are helpful to reduce PTSD symptoms is supported by promising experiences with approaches that include mindfulness and acceptance exercises in PTSD treatment, such as dialectical behavior therapy for PTSD (Steil et al., 2011; Bohus et al., 2013) and acceptance and commitment therapy (ACT; Twohig, 2009; Woidneck et al., 2014).

Furthermore, an increasing number of studies have analyzed mindfulness-based techniques as primary interventions. Two systematic reviews address the effects of mindfulness-based interventions (Banks et al., 2015) and meditation (Hilton et al., 2016) on PTSD symptoms. These reviews include different techniques, such as mindfulness-based stress reduction (MBSR; e.g., Kearney et al., 2013), mindfulness-based cognitive therapy (King et al., 2013), yoga (e.g., van der Kolk et al., 2014), and a mantram repetition program (e.g., Borman et al., 2013). Overall, the evidence in these studies supports the efficacy of mindfulness and meditation in reducing PTSD symptoms. Nevertheless, this evidence is limited due to the methodological deficits of most studies (Banks et al., 2015; Hilton et al., 2016).

Mindfulness-based stress reduction (MBSR; Kabat-Zinn, 2013) is currently the most frequently used mindfulness-based program in the treatment of PTSD. The pre-post effect sizes of MBSR on PTSD symptoms range from *d* = 0.55 for veterans (Kearney et al., 2012) to *d* = 1.54 for women with probable PTSD after interpersonal violence (Smith, 2010). In most MBSR studies, the participants were veterans (e.g., Kearney et al., 2013; Felleman et al., 2016), which limits the generalizability of the results. Another problem of existing studies is that patients were often allowed to participate simultaneously in more comprehensive psychological treatment (e.g., Kearney et al., 2013; Felleman et al., 2016), which impedes clear conclusions regarding the unique effects of mindfulness interventions. Furthermore, many studies did not confirm participants' PTSD diagnosis through a clinical interview (e.g., Kimbrough et al., 2010; Kearney et al., 2012; Felleman et al., 2016), and consequently, these studies often address symptoms in the subclinical sector. Nevertheless, there is promising evidence from several studies that have used MBSR successfully for veterans with a secured PTSD diagnosis (Bhatnagar et al., 2013; Kearney et al., 2013; Omidi et al., 2013). Furthermore, in a randomized controlled trial (RCT) also with veterans, MBSR has proven to be superior in reducing PTSD symptoms compared with and present-centered group therapy (Polusny et al., 2015). This large RCT overcomes many of the previously mentioned methodological deficits because it demonstrates the efficacy of MBSR in veterans with full PTSD who were not allowed to receive other simultaneous psychological treatment.

However, a new systemic review on emerging interventions for PTSD determines that the quality of the studies that assess MBSR as a treatment for PTSD still remains very low because of the described limitations of many studies (Metcalf et al., 2016). An additional problem in the application of MBSR to treat PTSD is that studies often report high dropout rates, e.g., from 22.4% in the newer RCT by Polusny et al. (2015) to 49% in a study concerning victims of interpersonal violence (Smith, 2010). Furthermore, many studies define completion as attending four or more sessions, which is a very low dose of treatment, and have percentages of non-completers that vary from 11 to 26% (Kimbrough et al., 2010; Kearney et al., 2012).

To sum up, there is promising evidence that mindfulness-based interventions, such as MBSR are effective in reducing PTSD symptoms. Until now, studies have primarily addressed veterans with war-related trauma. Moreover, these studies have mostly included patients who have experienced traumatic events but do not necessarily suffer from full PTSD, and they have often allowed other psychological interventions concurrently.

The present pilot study therefore aimed to test the feasibility of MBSR in patients who fully met a PTSD diagnosis after they experienced mixed traumatic events. In this study, MBSR was used as a standalone psychological intervention, which means that psychiatric medication was allowed but no simultaneous psychological treatments were allowed. The novelty of this study compared with former studies is that it combines both quantitative and qualitative methods to analyze the treatment effects and patients' subjective experiences with the program.

We hypothesized that there would be a significant reduction in PTSD and depressive symptoms (as evidenced by changes between pre-treatment scores, post-treatment scores and scores at 1-month follow-up) as well as an increase in mindfulness skills. To address the high dropout rates reported in former studies, we used a short questionnaire constructed to evaluate the applicability and the helpfulness of the program. Additionally, we carried out qualitative interviews with patients after the intervention. The aims of the interviews were to explore participants' subjective experiences and potential difficulties with the program, to analyze the psychological effects of the intervention and to collect ideas about how the specific needs of PTSD patients might be better addressed within a mindfulness-based treatment program.

## Materials and methods

### Procedure

Patients were recruited from the waitlist of our specialized PTSD outpatient center and an information center for victims of interpersonal violence. Those who seemed eligible for the study were contacted by phone, informed about the study, and if interested, invited to two assessment sessions, where inclusion and exclusion criteria were checked.

The inclusion criteria were a DSM-IV (American Psychiatric Association, 1994) diagnosis of PTSD, as determined by the Clinician-Administered PTSD Scale (Blake et al., 1995; Schnyder and Moergeli, 2002), age between 18 and 65 years, informed consent and the willingness to participate in the weekly sessions and to perform the corresponding exercises at home. Apart from meeting the trauma criterion of the DSM-IV, there were no requirements regarding the type of traumatic event the person had experienced.

The exclusion criteria were currently receiving a psychotherapeutic intervention as well as a lifetime diagnosis of schizophrenia, schizoaffective disorder or bipolar disorder according to the DSM-IV. Furthermore, we excluded patients who required treatment in another setting, such as those with acute suicide plans, a very low body mass index (<16) or current substance addiction. Furthermore, patients who were physically too restricted to participate in longer meditation or yoga exercises were excluded. After the assessment sessions, eligible patients participated in a short interview with the MBSR instructor to determine their motivation to regularly perform mindfulness exercises.

**Figure 1:**
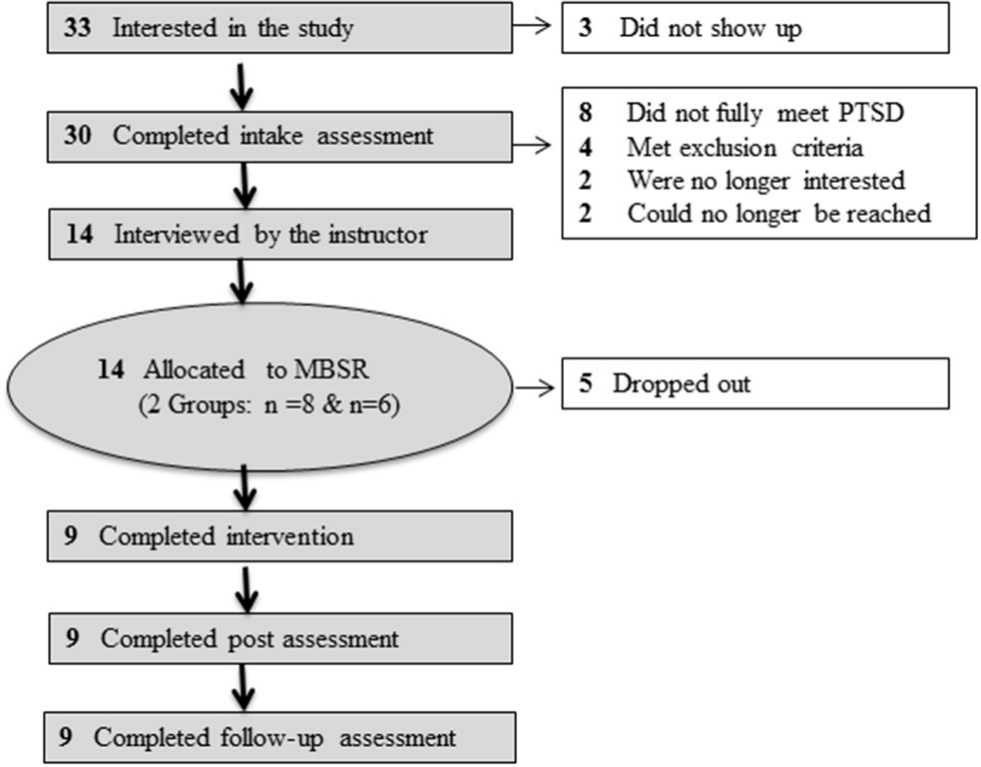
Patient flow.

Figure 1 describes the patient flow through the study. A total of 33 patients were interested in the study. Three did not show up for the first assessment session. Eight patients were excluded because they did not fully meet the PTSD diagnosis criteria in the Clinician-Administered PTSD Scale. Four met exclusion criteria (e.g., psychotic disorder). Two patients were no longer interested after the assessment sessions; one found the assessments very distressing, and one had already found another treatment option. Furthermore, two could no longer be reached by phone after the assessment sessions. Nobody was excluded after the interview with the MBSR instructor.

Fourteen participants who met the eligibility criteria and gave informed consent participated in two groups of 8-week MBSR sessions that started consecutively. The first group consisted of eight patients, and the second group consisted of six patients. The outcomes were assessed prior to treatment, post-treatment and at 1-month follow-up by trained clinical psychotherapists. Furthermore, directly after the intervention, participants were invited to participate in an interview (lasting up to 1 h) regarding their experiences with the MBSR program, which was carried out by a master's student. The patients who participated in more than half of the sessions and who did not drop out of the study were classified as completers.

### Participants

In total, 10 women and four men with an average age of 47.14 years (*SD* = 11.31; range 28–60 years) participated in this study. Six (43%) held a university degree, three (21%) held a high school degree, and five (36%) had finished secondary school as their highest education level. Five (36%) were currently incapable of working or were retired because of health reasons.

The participants suffered from PTSD after a diversity of events according to the A-criterion of the DSM-IV, e.g., a car accident, childhood sexual abuse, childhood physical abuse or a fire. Prior to treatment, the average duration of PTSD was 14.47 years (*SD* = 12.91; range 2 months–38 years). The average number of DSM-IV Axis-I disorders was 2.6 (*SD* = 1.16; range 1–5). The most frequent comorbid diagnoses were affective disorders (86%) and other anxiety disorders (21%). Thirteen patients had received prior outpatient treatment (93%), and five of these patients had also received inpatient treatment (36%). Seven (50%) received psychopharmacological medication and were asked not to change medication until follow-up assessments.

### The intervention

A certified MBSR instructor without experience in treating PTSD guided the intervention in accordance with the common standards for MBSR, which consists of eight weekly group sessions (2.5 h each) and a daylong retreat (Kabat-Zinn, 2013). The aims of the program are to train mindfulness through formal practice and to establish present-moment awareness in daily activities, such as eating and stair climbing. MBSR includes practical exercises, information about stress and its consequences, and an exchange regarding the daily exercises the participants perform between sessions. The formal practice consists of a body scan, yoga, and sitting and walking meditation. During the body scan, the attention is directed systematically and step by step through various areas of the body. The aim of this exercise is a mindful and non-judgmental awareness of the body in the present moment. The yoga exercises come from hatha yoga and involve performing movements with present-moment attention. During sitting meditation, which begins with short sitting periods that are increased incrementally to 30-min meditations, the focus is on the breath. Walking meditations differ from daily walking in terms of the absence of a concrete goal and the direction of attention to the walking itself. The daylong retreat that took place at the end of the course lasted 8 h and offered the opportunity to deepen the practice. During all sessions, an experienced PTSD therapist was present to intervene in cases of acute crisis. For the same reason, patients also received her phone number and were allowed to call her between the sessions.

### Measures

*Structured Clinical Interview for the DSM*-IV (*SCID-I*; German Version: Wittchen et al., 1997). The SCID-I was used to assess Axis-I diagnoses before treatment and to check for inclusion and exclusion criteria.

*Clinician-Administered PTSD Scale* (*CAPS*; Blake et al., 1995; Schnyder and Moergeli, 2002). We used the semi-structured CAPS interview, which measures the frequency and intensity of 17 PTSD symptoms over the past 4 weeks at baseline and follow-up. After traumatic events were identified with the *Life Event Checklist* (*LEC*; Gray et al., 2004) at baseline, the symptoms regarding the three most distressing events (e.g., sexual abuse by the stepfather) were defined as the focus of the interview. We used the recommended scoring rule that requires a frequency score of 1 and an intensity score of 2 to consider a symptom to be fulfilled (Weathers et al., 1999). A severity score was built by summing up the frequency and intensity scores for the 17 symptoms (range 0–136).

*Davidson Trauma Scale (DTS;* Davidson et al., 1997). The DTS is a 17-item self-rating scale that assesses the frequency and severity of PTSD symptoms within the past week on a 5-point scale ranging from 0 to 4. The sum score ranges from 0 to 136. In the present study, Cronbach's alpha was 0.94 at baseline. The DTS was administered at each time point.

*Beck Depression Inventory (BDI-II*; Beck et al., 1996; Hautzinger et al., 2006). The BDI is a 21-item questionnaire that measures depressive symptoms over the past week. In the present study, Cronbach's alpha was 0.87 at baseline. The BDI was administered at each time point.

*Five Facet Mindfulness Questionnaire (FFMQ;* Baer et al., 2006). The FFMQ assesses five skills of mindfulness: “observing,” “describing,” “acting with awareness,” “non-judging of inner experience,” and “non-reactivity to inner experience.” These five skills have turned out to be crucial (Baer et al., 2006). In the present study, Cronbach's alpha for the full scale was 0.79 at baseline. The FFMQ was administered at baseline and follow-up.

*Questionnaire to evaluate the program (QeP)*. Constructed for the purpose of this study, the QeP is a short questionnaire for PTSD patients to evaluate the MBSR program. It consists of nine items regarding the individually experienced applicability and helpfulness of the program in general and the different exercises (body scan, sitting mediation, walking mediation, yoga, mindfulness in daily activities) in particular, e.g., “How applicable were the exercises?” and “How helpful was the sitting mediation?” Two further questions address the usefulness of the program to improve patients' management of acute crisis and to reduce PTSD symptoms. Every question is rated on a 5-point Likert scale ranging, e.g., from “not useful” to “very useful.”

### Qualitative interviews

The qualitative interviews took place the week after the intervention. The aims were to explore participants' experiences with the program, to analyze the underlying mechanisms of psychic improvements and to gather patients' ideas to better adapt the program to the specific needs of PTSD patients. The interviews were semi-structured and guideline based and followed an explorative approach. They started with an open question (“What comes up in your mind when you think about the mindfulness program?”) and then focused on participants' subjective experience and potential difficulties with the exercises (e.g., “What was your experience with the single exercises?”), within the group (e.g., “How did you feel in the group?”) and with the instructor. The next part of the interview focused on the psychological effects of the intervention (e.g., “What changes in your psychic state have you noticed since your attendance at the MBSR course?”). In the last part of the interview, participants were asked for their feedback regarding the program and their ideas for future improvement.

### Data analysis

#### Statistical analysis

Single missing items were substituted with multiple imputation, in which one complete dataset is calculated. Imputation in SPSS is based on a Markov chain Monte Carlo algorithm (MCMC; Little and Rubin, 2002).

To evaluate the effects of the program on PTSD, depressive symptoms and mindfulness skills, hierarchical linear models [with two levels: observations (ratings) were clustered in participants] were used to determine the changes in symptoms from baseline to post-treatment and follow-up. These models allow for individual intercepts of the patients that represent the different baseline values and an autoregressive structure, which has a good fit in repeated measures models. The analyses were conducted on an intention-to-treat (ITT) basis. For dropouts, we used the last observation carried forward technique in which the last observed value is substituted for all of a patient's subsequent missing values.

Treatment effect sizes were calculated for the entire sample (ITT based on the last observation carried forward for the dropouts) and for the completers by using Cohen's *d*: effect sizes were defined as small (*d* = 0.20), medium (*d* = 0.50), and large (*d* = 0.80; Cohen, 1988). A bivariate correlation analysis was performed to determine whether an augmentation of mindfulness skills was associated with a reduction in PTSD and depressive symptoms in the completers.

Furthermore, in an explorative analysis, *t*-tests were conducted to check for differences between the completers and the dropouts regarding PTSD and depression symptom severity as well as mindfulness skills at baseline. Through *t*-tests and Chi-square tests, the two groups were also compared regarding socioeconomic data, the number of comorbid diagnoses and the duration of PTSD symptoms.

#### Analysis of the qualitative interviews

The interviews were recorded, transcribed and entered into the computer software MAXQDA 2007 to support the data handling (Kuckartz, 2007). Afterwards, the interviews were coded, and their content was analyzed. To develop a system of categories, as a first step, the material of one interview was categorized inductively (Charmaz, 2006) by two raters sticking closely to the raw data. In a second step, categorizing was augmented by deductive methods (Mayring, 2010) by including relevant themes from the interview guidelines (e.g., suggestions for improvement) in the system of categories in order to be able to answer the previously defined research questions. The system of categories was completed while coding the subsequent interviews. Intercoder reliability was calculated using Cohen's kappa (Cohen, 1960). For this purpose, one interview was selected at random. This interview corresponded to approximately 10% of the data material, as proposed by Neuendorf (2002). One rater coded the interview and marked the text segments he had coded. Then, the second rater coded the marked segments independently from the first rater. After determining inter-coder reliability, one rater analyzed the remaining interviews; in cases of uncertainty, he consulted the second rater. The ratings were performed by two master's students supervised by the first author.

## Results

Five patients (35.71%) dropped out after an average of 2.6 sessions (*SD* = 1.14; range 1–4). Nine patients were classified as completers, which means that they participated in more than 50% of the sessions. All completers participated in the post-treatment and follow-up assessments. The completers attended an average of 6.89 sessions (*SD* = 1.17; range 5–8) and reported that they had practiced the learned techniques an average of 5.06 times per week (*SD* = 0.85; range 3.5–6). During the sessions, no acute crisis occurred. Between sessions, the PTSD therapist received one call from a patient who felt considerable pressure regarding the yoga exercises.

### Quantitative analysis

The hierarchical linear model indicated a significant reduction in PTSD symptoms, as measured with the CAPS [*t*_(13)_ = −2.70; *p* = 0.018]. The ITT effect sizes from baseline to follow-up were in the medium range, and for the completers, they were large (see Table 1). At follow-up, six of the nine completers no longer fully met PTSD diagnostic criteria, according to the CAPS interview. One patient exhibited a deterioration in the CAPS from baseline (mean score = 62) to follow-up (mean score = 83). In the hierarchical linear models regarding self-reported PTSD symptoms, as measured with the DTS, there were also significant reductions [frequency: *t*_(29.78)_ = −2.67; *p* = 0.012; severity: *t*_(29.50)_ = −2.25; *p* = 0.032]. The ITT effect sizes from baseline to follow-up were in the medium range, and for the completers, they were medium to large (see Table 1).

**Table 1:** Descriptive statistics and effect sizes for completers (*n* = 9) and intention to treat (*n* = 14).

	**Pre Mean (SD)**	**Post Mean (SD)**	**FU Mean (SD)**	**Pre-post effect sizes^a^**	**Pre-FU effect sizes^a^**
**COMPLETERSs**					
**CAPS**					
Total score	68.72 (12.46)		44.00 (26.02)		1.20
Intrusions	22.78 (4.89)		12.22 (9.12)		1.40
Avoidance	25.83 (8.47)		15.89 (11.90)		1.00
Arousal	20.11 (5.56)		15.89 (7.70)		0.60
**DTS**					
Total score	80.56 (23.63)	67.33 (31.22)	60.78 (36.23)	0.5	0.6
Frequency	40.67 (11.05)	33.22 (14.80)	30.56 (16.93)	0.6	0.7
Severity	39.89 (13.61)	34.11(16.91)	30.22(19.72)	0.4	0.6
**BDI-II**					
Total score	31.56 (10.04)	28.00(15.94)	25.00 (16.38)	0.30	0.50
**FFMQ**					
Mean score	2.82 (0.41)		3.00 (0.67)		0.30
Non-judging	3.18 (0.74)		3.33 (0.86)		0.20
Observing	2.90 (0.67)		3.17 (0.92)		0.30
Non-reactivity	2.44 (0.50)		2.63 (0.77)		0.30
Describing	3.04 (0.80)		3.24 (0.98)		0.20
Acting with awareness	2.47 (0.60)		2.63 (0.95)		0.20
**INTENTION TO TREAT**					
**CAPS**					
Total score	75.25 (15.37)		59.36 (30.53)		0.7
Intrusions	23.14 (5.01)		16.36 (9.72)		0.9
Avoidance	30.11 (9.56)		23.71 (14.76)		0.5
Arousal	22.00 (6.54)		19.29 (8.70)		0.4
**DTS**					
Total score	78.21 (26.96)	69.71 (31.36)	65.50 (34.98)	0.3	0.4
Frequency	38.86 (12.64)	34.07 (14.64)	32.36 (16.15)	0.4	0.4
Severity	39.36 (15.06)	35.64 (17.12)	33.14 (19.19)	0.2	0.4
**BDI-II**					
Total score	32.71 (10.68)	30.43 (14.74)	28.50 (15.44)	0.2	0.3
**FFMQ**					
Mean score	2.85 (0.35)		2.97 (0.55)		0.3
Non-judging	3.05 (0.74)		3.13 (0.80)		0.1
Observing	3.03 (0.74)		3.23 (0.83)		0.3
Non-reactivity	2.40 (0.47)		2.50 (0.63)		0.2
Describing	3.17 (0.91)		3.24 (0.99)		0.1
Acting with awareness	2.55 (0.51)		2.63 (0.72)		0.1

*CAPS, Clinician-Administered PTSD Scale; DTS; Davidson Trauma Scale; BDI-II, Beck Depression Inventory; FFMQ, Five Facet Mindfulness Questionnaire*.

a *Cohen's d*.

Furthermore, there was a trend toward a reduction in depressive symptoms, as measured with the BDI [*t*_(29.77)_ = −1.94; *p* = 0.063]. Regarding the mindfulness skills that were measured with the FFMQ, there was no statistically significant increase, but a trend toward improved “observing” [*t*_(13)_ = 1.98; *p* = 0.070].

A correlational analysis revealed a trend toward a significant association between an increase in mindfulness skills and a decrease in PTSD symptoms, as measured with the CAPS [*r*_(8)_ = 0.54; *p* = 0.085], and depression, as measured with the BDI [*r*_(8)_ = 0.61; *p* = 0.053].

In the QeP, 66.6% of the completers evaluated the exercises as “applicable” or “very applicable,” and 33.3% rated the exercises as “partly applicable.” Table 2 shows the evaluations of the helpfulness of mindfulness techniques in general and the helpfulness of the different exercises in particular.

**Table 2:** Evaluation of the different exercises.

	**Not helpful %**	**A little helpful %**	**Partly helpful %**	**Mainly helpful %**	**Very helpful %**
Mindfulness techniques overall (*n* = 9)	/	/	22.2	55.6	22.2
Body scan (*n* = 9)	11.1	11.1	11.1	44.4	22.2
Sitting meditation (*n* = 9)	/	/	22.2	33.3	44.4
Walking meditation (*n* = 6)	16.7	16.7	16.7	33.3	16.7
Yoga (*n* = 9)	11.1	33.3	11.1	11.1	33.3
Mindfulness in daily activities (*n* = 9)	/	/	33.3	33.3	33.3

Regarding the usefulness of the program to improve their management of acute crisis, 44.4% of completers evaluated the program as “very useful” or “mainly useful,” and 55.6% evaluated it as “partly useful.” Furthermore, with respect to its usefulness in reducing PTSD symptoms, 44.4% evaluated the program as “mainly useful,” and 44.4% evaluated it as “partly useful.” Overall, 11.1% rated the program as “a little useful.”

In the *t*-tests performed to analyze the differences between completers and dropouts regarding symptom severity, a significant difference in the CAPS was found. The dropouts reported significantly more PTSD symptoms at baseline than the completers [*t*_(12)_ = −2.51, *p* = 0.026]; see Table 3. In the DTS, the BDI and the FFMQ, no significant differences were found. Furthermore, the dropouts and completers did not differ regarding the number of diagnoses or PTSD symptom duration. Additionally, regarding the socioeconomic data (gender, age and education level), no significant differences were found.

**Table 3:** Comparison of baseline characteristics between completers and dropouts: means, standard deviations and effect sizes.

	**Completers (*n* = 9) Mean (SD)**	**Dropouts (*n* = 5) Mean (SD)**	**Effect sizes^a^**
CAPS (total score)	68.72 (12.46)	87.00 (13.73)	1.4
DTS (total score)	80.56 (23.63)	74.00 (34.79)	0.2
BDI-II (total score)	31.56 (10.04)	34.80 (12.78)	0.3
FFMQ (mean score)	2.82 (0.41)	2.91 (0.24)	0.3
Number of diagnoses	2.89 (1.37)	2.00 (0.71)	0.8
PTSD symptom duration in years	15.15 (14.35)	12.67 (10.26)	0.2

*CAPS, Clinician-Administered PTSD Scale; DTS, Davidson Trauma Scale; BDI-II, Beck Depression Inventory; FFMQ, Five Facet Mindfulness Questionnaire*.

a *Cohen's d*.

### Results of the qualitative interviews

All nine completers and one patient who dropped out participated in the qualitative interviews. The average duration of the interviews was 38 min (*SD* = 5.38; range 29–46). The Cohen's kappa was 0.75, which indicated good agreement between the raters (Landis and Koch, 1977).

#### Experiences with the exercises

Most participants judged the intervention and the corresponding exercises overall as positive and helpful, e.g., they described the intervention as “*strengthening*” (Patient 3), “*something that makes sense*” (Patient 11) and “*the right way*” (Patient 7). Furthermore, participants reported that it had been helpful that they did not have to talk about their trauma in the group (e.g., Patient 5) but were encouraged to allow the corresponding pain and suffering to be part of their experiences.

The regularity and the taught discipline regarding the exercises in the group and at home were on the one hand considered helpful but on the other hand also experienced as “*rigid*” (Patient 12) and “*not personalized*” (Patient 7), with too little time for individual questions (Patient 6). Furthermore, some patients explained that particularly at the beginning, the intervention had been “*incredibly difficult*” (Patient 7) and led to feelings of “*overextension, exhaustion and desperateness*” (Patient 6). Some participants criticized that exercises had been taught too fast and with little explanations regarding their aims (e.g., Patient 4).

The formal practices, particularly the body scan and sitting meditation, were considered overall to be “*pleasing*” (Patient 4) and “*helpful*” (Patient 12). Reported positive aspects of the exercises included “*concentration on oneself*” (Patient 5) and the “*ability to distance oneself from distressing experiences”* (Patient 10). They were associated with silence (Patient 2) and concentration on inner processes (Patient 12). Nevertheless, there were patients who characterized the exercises as “*difficult to concentrate on*” (Patient 11), “*difficult to remember*” (Patient 4) and “*time consuming*” (Patient 7). Furthermore, various patients (e.g., Patients 4 and 1) mentioned that these exercises could not be applied in acute crises.

The yoga exercises were considered “*relaxing*” by some patients (e.g., Patient 2) and helpful due to the concentration on the movements (Patient 12). Two patients reported fundamental problems: “*Yoga is definitely not my thing*” (Patient 1) and “*I did not get acquainted with the exercises*” (Patient 5). Furthermore, three patients reported difficulties with the implementation due to physical problems (Patients 1, 4, and 10), and some patients criticized that the instructor did not demonstrate the exercises, which led them to worry that they were doing something wrong (e.g., Patient 3).

The establishment of mindfulness in daily activities was considered “*positive in every respect*” (Patient 12). The patients mentioned that it had been helpful to “*concentrate on the present moment, with everything that is part of it*” (Patient 5) and to be more aware of positive things (e.g., Patient 3), and they stated that the mindful execution of daily tasks had a “*meditative character*” (Patient 2).

#### Experiences with the group, the MBSR trainer and the trauma therapist

Participants perceived the group atmosphere as quite diverse. Positive impressions ranged from a “*quiet, nice atmosphere*” with a helpful exchange (Patient 5) to “*good to know that everybody carries his/her bag*” (Patient 3). Those who perceived the group more negatively described the atmosphere as “*depressed*” (Patient 1), with many people appearing to be fearful and incommunicative (Patient 4). One patient mentioned that particularly at the beginning of the course, she had wanted to “*be left alone*” (Patient 6), and others described that they had felt “*isolated*” (Patient 7) due to the missing cohesion (e.g., Patient 11) and a lack of exchange (e.g., Patient 9).

The MBSR instructor was classified by some patients as “*helpful*” (Patient 6), as a “*coach*” (Patient 5), and as “*doing a good job*” (Patient 2). However, the participants also mentioned several critical points regarding the way she addressed PTSD symptoms, such as nightmares (Patient 7) and her teaching style, which according to some patients, impeded the establishment of a connection to all participants (Patient 12) and to give them orientation (Patient 1).

The presence of the PTSD therapist was overall considered positive. She was described as “*very positive and motivating*” (Patient 3) and as “*someone who can establish a connection*” (Patient 12). Furthermore, patients assessed the knowledge that they were allowed to call her in moments of acute crisis as very helpful (e.g., Patients 3 and 7).

#### Effects of the intervention

The majority of participants reported a general augmentation of wellbeing, which was associated with an increased awareness of positive things (e.g., Patients 1 and 10), an increased experience of positive emotions (e.g., Patients 5 and 7) and a reduction in negative thoughts (Patient 11). Additionally, some patients mentioned caring more for themselves (e.g., Patients 10 and 12) and being able to make plans for the future again (Patient 4). Furthermore, an augmentation of performance in private and professional contexts was observed (e.g., Patients 4 and 5) as well as a reduction in sleep disturbances (Patients 2 and 4) and better management of physical pain (Patient 7).

More precisely, changes in handling difficult situations, particularly negative thoughts and feelings, were observed (e.g., Patients 5 and 11). In this context, patients also reported a better ability to distance themselves from stress (e.g., Patients 5 and 11) and anger (Patient 10), a reduction in irritability (“*I don't explode anymore*,” Patient 2) and faster stabilization in difficult situations (Patient 7). Furthermore, one participant mentioned being able to prioritize upcoming tasks more easily (Patient 3).

Another important area of change for many of the participants were trauma-related symptoms: the patients reported a reduction in trauma-related thoughts (Patients 4 and 11) and an improved handling of trauma-associated memories (e.g., Patients 3 and 4). For example, for one patient, these memories had “*lost their dread*” (Patient 12), and she knew that they would disappear again. Additionally, after the intervention, the participants more often considered the traumatic event as part of the past (e.g., Patients 2 and 4) and were able to reduce avoidance (Patients 1 and 5), e.g., by talking again about issues that were associated with the trauma (Patient 5). Regarding trauma-related cognitions, one patient reported that it was easier to not judge the traumatic event and to not take it personally, which resulted in the impression of not being “*involved that much anymore*” (Patient 7). Additionally, three patients reported that their acceptance of the traumatic event as a part of their lives had been augmented and that they had stopped struggling with it (Patients 3, 4, and 12), which for one patient, was associated with a reduced condemnation of herself for her symptoms (Patient 12).

One patient reported an aggravation of her psychological symptoms, which in her opinion was probably related to a deterioration of a physical disease and not to the intervention (Patient 10). Another participant described her psychological status as “*fluctuating between ups and downs*” (Patient 1). Alternating with good days, she described situations in which she had the impression of “*falling even deeper*” than before, which in her opinion might be a side effect of the intervention (Patient 1).

#### Ideas for improvement

Many patients claimed more transparency regarding the aims of the program, the single exercises and the dropouts (e.g., Patients 4 and 7). One participant proposed starting each session with an overview of the time structure and the planned practices (Patient 2). Furthermore, many patients requested more concrete instructions regarding the exercises, particularly the yoga exercises (e.g., Patients 4 and 6), combined with a demonstration of those by the instructor (e.g., Patients 3 and 5). Furthermore, some participants expressed the wish to reflect more about their experiences with the exercises (e.g., Patient 12), particularly the homework assignments (Patients 4 and 12), and one proposed performing the intervention in a more individual setting (Patient 5).

Due to concentration difficulties, one patient requested reducing the duration of the formal exercises to 30 min (Patient 11), while another one proposed including “*shorter but more intense*” exercises that could be used in difficult situations (Patient 1). Furthermore, one patient would have preferred an intervention without yoga exercises (Patient 1).

Regarding group cohesion, some participants expressed the desire to get to know one another better (Patient 2) and to exchange more ideas regarding their experiences with the exercises (e.g., Patients 4 and 10). In the opinion of some participants, the instructor should be more “*friendly*” and “*facilitate feelings of welcomeness*” (Patient 7). Furthermore, the instructor should motivate the participants more (Patient 11) and give more positive feedback (Patient 12). In cases of trauma-related reports by a group member, the instructor should be able to “*manage this very well*” (Patient 7). In this context, stronger involvement on the part of the trauma therapist was suggested (Patient 7).

## Discussion

In this pre-post study, we tested the feasibility of an 8-week MBSR as a standalone group intervention (apart from medication) for patients with PTSD who have experienced different types of traumatic events. The efficacy of the intervention was analyzed by quantitative data. Furthermore, qualitative interviews were used to explore patients' experiences with the exercises to better understand the effects of the intervention and to gather ideas for improvement.

After the intervention, six of the nine completers no longer fully met the criteria for PTSD diagnosis (42.9% of the original sample), which is especially remarkable because it was a chronic sample with an average duration of almost 15 years of PTSD. Overall, our qualitative results confirm that MBSR as a standalone mindfulness-based intervention can be used to reduce PTSD and depression in a sample of patients with PTSD after mixed traumas. The effect sizes were similar to those found in previous studies (e.g., Kimbrough et al., 2010; Kearney et al., 2012). In the post-intervention interviews and the questionnaire to evaluate the program, the majority of the participants assessed the exercises as applicable and helpful, which was particularly true for the sitting meditation, the body scan and the implementation of mindfulness in daily activities.

The results of the qualitative interviews contribute to a better understanding of the effects of the intervention: the majority of patients reported an augmentation of wellbeing, which, among other factors, was associated with an increase in positive emotions, better performance and more self-care. Furthermore, changes were reported regarding the handling of difficult situations, which was associated with, e.g., a greater inner distance from these situations and a reduction in irritability. After the intervention, improvements regarding all four PTSD symptom clusters (intrusion, avoidance, negative alterations in cognitions and mood, and alterations in arousal and reactivity) were reported. Overall, the patients felt more distant from the traumatic event and accepted it more as part of their personal history. Correspondingly, a reduction in trauma-related thoughts and negative cognition and a decrease in avoidance behavior were reported, which is consistent with previous studies that demonstrated particularly strong effects of mindfulness on avoidance symptoms (Banks et al., 2015).

Despite these promising results, it must be considered that one patient deteriorated in the CAPS, and similar to former studies (e.g., Smith, 2010; Kearney et al., 2012), approximately one-third of the participants dropped out and did not complete further assessments. As a consequence, it remains unclear whether those patients would have benefitted from the intervention. The only difference that was found between the dropouts and completers was a greater PTSD symptom severity at baseline, which is particular remarkable because a higher baseline PTSD has been shown to predict a greater reduction of symptoms through MBSR (Felleman et al., 2016). Thus, it may be especially important to find ways to keep severely affected patients from dropping out.

The results of the qualitative post interviews suggest possible difficulties that PTSD patients may have with MBSR. First, some patients perceived the intervention as too rigid and not personalized. They also criticized that there had been too little explanation regarding the exercises and their benefit. Furthermore, some patients reported difficulty concentrating on and remembering the exercises, and they mentioned that they were not applicable in acute crisis. Correspondingly, in the questionnaire to evaluate the program, despite the high levels of acceptance, more than one third of patients assessed the exercises as only partly applicable. In particular, many of the participants reported having difficulties with the yoga exercises, which were associated with the lack of a demonstration of these exercises and with physical problems that are often found among PTSD patients due to an increased prevalence of physical diseases (Spitzer et al., 2009). Furthermore, some patients reported that they had not felt comfortable in the group. In the opinion of some patients, the MBSR instructor had difficulties in addressing their specific needs, e.g., to make them feel secure. However, this may be different with another instructor who is better skilled at contacts with PTSD patients.

One idea to better adapt the intervention to the specific needs of PTSD patients that resulted from the qualitative interviews is to provide more information regarding the exercises and their utility to reduce PTSD, which has been successfully implemented in a “trauma-informed MBSR program” for survivors of interpersonal violence (Kelly and Garland, 2016). Such a greater transparency may give patients a greater sense of control, which is particularly important because of the strong loss of control that is suffered during a traumatic event.

Regarding the distinct benefit of the different formal exercises, dismantling studies are needed. The mainly positive evaluations of the sitting meditation and body scan in our study are in line with a study that demonstrated both of these exercises to be superior to slow breathing and sitting quietly (Colgan et al., 2016). Whether yoga really is unsuitable for PTSD patients, as our qualitative results may suggest, or the reported problems resulted from the teaching style of this specific instructor is difficult to answer. Support for the idea that the teaching style may be the problem comes from studies that demonstrated yoga to be applicable and helpful to PTSD sufferers (van der Kolk et al., 2014). Due to the concentration difficulties that are typical in PTSD (American Psychiatric Association., 2013), it may be considered to allow a more flexible adaptation of the exercises or to reduce their duration, which at least has been found not to limit their efficacy in patients with chronic pain (Sagula, 1999). Furthermore, as proposed by some patients, shorter exercises that could help to manage acute distress, e.g., when confronting trauma-related triggers, may be a valuable addition to standard MBSR.

Regarding the reported lack of group cohesion, it is important to consider that in groups of PTSD patients, who often have lost confidence in others, it is a key task of the therapist to establish trustful interactions among the group members (Knaevelsrud et al., 2012). Because of the problems that were reported regarding the group setting, in addition to considering the proposed adaptions to standard MBSR in groups, an individual treatment setting may be considered as an alternative. Arguments for an individual treatment setting may be that individual treatment for PTSD has been proven to be superior to group interventions (Taylor and Harvey, 2009) and that dropout rates are usually lower in individual treatment (Imel et al., 2013). However, the benefits of group settings, such as the exchanges among patients, would be lost. Finally, independent of the setting, it would be helpful for instructors to be skilled in working with PTSD patients, beyond having knowledge of mindfulness.

### Limitations

The results of this study must be interpreted with caution due to several limitations. First, the generalizability is limited due to the small sample size, the lack of a control group and the fact that most of the patients suffered from long-term PTSD and the results may thus apply specifically to chronic PTSD. Furthermore, only one MBSR instructor participated in this study. Therefore, it remains unclear whether the problems reported depend on the suitability of MBSR for this patient group or also on the instructor's teaching style. In addition, the fact that only one patient who dropped out participated in the qualitative post interviews further limits their validity. More studies, particularly RCTs, are needed to further investigate the efficacy of standalone mindfulness interventions for PTSD. Future studies should also analyze the appropriateness and effectiveness of particular exercises, such as the body scan, sitting mediation and yoga, for PTSD sufferers and develop interventions that are tailored to the specific needs of this patient group.

## Conclusion

This study contributes evidence that standalone mindfulness-based interventions seem to be applicable and helpful for patients with PTSD who have experienced different types of traumatic events. However, the high dropout rates and the results of the post-intervention interviews suggest that the intervention should be better adapted to the specific needs of PTSD patients. Considerable options for a better adaptation are: including psychoeducation, providing more information regarding the aims of the exercises, allowing a more flexible adaptation of the experiences, using shorter exercises to manage acute distress and perhaps an individual treatment setting.

## Ethics statement

This study was carried out in accordance with the recommendations of ethics committee of Goethe University, Frankfurt am Main. All subjects gave written informed consent in accordance with the Declaration of Helsinki. The protocol was approved by the ethics committee of Goethe University.

## Author contributions

MM, RS, and SW conceptualized the study. MM and MV collected, processed and analyzed the data. MM prepared the manuscript. SW, MV, and RS critically revised and edited the manuscript.

### Conflict of interest statement

The authors declare that the research was conducted in the absence of any commercial or financial relationships that could be construed as a potential conflict of interest.
